# Targeting Cancer Cells Overexpressing Folate Receptors with New Terpolymer-Based Nanocapsules: Toward a Novel Targeted DNA Delivery System for Cancer Therapy

**DOI:** 10.3390/biomedicines9091275

**Published:** 2021-09-21

**Authors:** Elena Bellotti, Maria Grazia Cascone, Niccoletta Barbani, Daniela Rossin, Raffaella Rastaldo, Claudia Giachino, Caterina Cristallini

**Affiliations:** 1 Department of Civil and Industrial Engineering, University of Pisa, 56122 Pisa, Italy; maria.grazia.cascone@unipi.it (M.G.C.); niccoletta.barbani@unipi.it (N.B.); 2 Department of Clinical and Biological Sciences, University of Turin, 10143 Turin, Italy; d.rossin@unito.it (D.R.); raffaella.rastaldo@unito.it (R.R.); claudia.giachino@unito.it (C.G.); 3 Institute for Chemical and Physical Processes, IPCF ss Pisa, 56122 Pisa, Italy

**Keywords:** polymeric nanoparticles, nanocapsules, gene therapy, active targeting, DNA delivery, folic acid, terpolymer, cancer

## Abstract

Chemotherapeutics represent the standard treatment for a wide range of cancers. However, these agents also affect healthy cells, thus leading to severe off-target effects. Given the non-selectivity of the commonly used drugs, any increase in the selective tumor tissue uptake would represent a significant improvement in cancer therapy. Recently, the use of gene therapy to completely remove the lesion and avoid the toxicity of chemotherapeutics has become a tendency in oncotherapy. Ideally, the genetic material must be safely transferred from the site of administration to the target cells, without involving healthy tissues. This can be achieved by encapsulating genes into non-viral carriers and modifying their surface with ligands with high selectivity and affinity for a relevant receptor on the target cells. Hence, in this work we evaluate the use of terpolymer-based nanocapsules for the targeted delivery of DNA toward cancer cells. The surface of the nanocapsules is decorated with folic acid to actively target the folate receptors overexpressed on a variety of cancer cells. The nanocapsules demonstrate a good ability of encapsulating and releasing DNA. Moreover, the presence of the targeting moieties on the surface of the nanocapsules favors cell uptake, opening up the possibility of more effective therapies.

## 1. Introduction

Chemotherapy is the major therapeutic approach for the treatment of a wide range of cancers. A major problem with such treatment is represented by side effects as the agents affect healthy tissues as well as cancerous cells [1,2,3]. Hence, any selective increase in tumor tissue uptake would be a significant improvement in cancer therapy given the non-selectivity of the commonly used drugs [4]. With the progress in molecular biology and biotechnology, most cancers have been discovered to be caused by genetic mutations [5,6,7,8,9,10]. Consequently, gene therapy has been acknowledged as major progress of modern medicine and a focus for oncotherapy research. In particular, the application of gene therapy to completely remove the lesion and avoid the serious overall toxicity and side effects becomes a tendency in the development of oncotherapy [11,12,13,14,15]. Specifically, in oncotherapy cancer cells can be modified with genes of cytotoxic or tumor suppressive proteins, or with a class of suicidal genes in combination with pro-drugs, all of which result in autonomous cell death [16]. Alternatively, the anti-tumor immune response can be elicited by genetic modification of malignant cells or immune cells to produce cytokines or tumor antigens. Generally, the genetic material must be safely transferred from the site of administration to the target cells. Depending on the nature of the carrier, there are two different approaches for gene delivery, consisting in viral and non-viral delivery [5,17,18,19,20]. In particular, viral vectors mediate gene transfer with high efficiency and can ensure long-term gene expression, however there are many safety concerns related to their use including vector antigenicity, inflammation, and possible insertional mutagenesis [5,21]. On the other hand, non-viral vectors have presented some crucial advantages over viral vectors to improve the toxicity and targeting problems by using nanotechnology in tumor tissues. These carriers include lipids, biomaterials, synthesized polymers, and dendrimers [22,23,24,25,26,27,28]. In all cases, the main goal is to select the target cells with a reduction in the number of non-cancer cells affected, thus reducing systemic side effects [29,30]. An example is represented by the active targeting, also called ligand-mediated targeting, by exploiting specific ligands on the surface of the non-viral carrier, with high selectivity and affinity for a relevant receptor on the target cells [31]. This approach is beneficial in terms of enhancing accumulation at the target site and decreasing the exposure of healthy cells to the drug. Active targeting has been efficiently exploited to increase nanoparticle internalization by target cells and improve the efficacy of their payloads [32,33,34,35,36].

Ligands are selected to bind surface molecules or receptors overexpressed in diseased organs, tissues, cells or subcellular domains. In the development of anti-tumor agents, including DNA, much attention has been given to systems targeting the folate receptors [37,38,39,40,41,42,43,44,45]. The folate receptor is an affinity membrane folate-binding protein which after folic acid binding trigger endocytosis with consequent cell uptake. Its expression is negligible in healthy cells while it is largely present in myeloid leukemia [46] and various solid tumors such as lung, brain, ovarian, prostate, breast, gastric and colorectal [47]. Over expression of folate receptors on cancer cells makes it a potential target [48]. For this reason, it has been exploited by diagnostic tools for bioimaging of tumor cells [49]. Moreover, advantages of a small targeting ligand like folic acid over larger entities are represented by limited changes in dimensions of the carrier and lower or absent antigenicity [50]. 

In this study we evaluated our previously developed and fully characterized terpolymer-based nanoparticles [51] to be used for targeted DNA delivery, using folic acid as ligand to obtain active targeting toward cancer cells. Nanoparticles were fabricated in the form of nanocapsules due to their increased ability to encapsulate and release the active principle of interest. 

## 2. Materials and Methods

All the materials and reagents were obtained from Sigma-Aldrich (Sigma-Aldrich, Milan, Italy) unless otherwise specified.

### 2.1. Preparation of Nanocapsules

Terpolymer-based nanocapsules (NCs) were fabricated following the same protocol described in our previous paper [51]. Briefly, NCs were obtained by radical polymerization starting from a diluted solution of butyl methacrylate (BMA, Mw: 142.2 g/mol, density: 0.894 g/cm^3^), poly(ethylene glycol) methyl ether methacrylate ((PEG)MEMA, Mw: 300 Da, density: 1.05 g/mL) and 2-(dimethylamino)ethyl methacrylate (DMAEMA, Mw: 157.21 g/mol, density: 0.933 g/mL), with 80/10/10 percentage molar ratio and in the presence of trimethylpropane trimethacrylate (TRIM) as cross-linker (20% mol/mol). The chemical structures of the monomers and cross-linker used for the synthesis are shown in Table 1. 

The polymerization was carried out around a preformed polymeric core. First, polymethyl methacrylate (PMMA)-based nanoparticles to be used as the sacrificial core were synthesized by emulsion polymerization of methyl methacrylate (MMA, Mw: 100.12 g/mol, density: 0.940 g/cm^3^) in the presence of sodium dodecyl sulfate (SDS, Mw: 288 g/mol) as surfactant. The polymerization was carried out in a mixture of water/ethanol (60/40 *v/v*) in the presence of sodium metabisulfite (Na_2_S_2_O_5_, Carlo Erba Reagenti, Milan, Italy) and ammonium persulfate ((NH_4_)_2_S_2_O_8_, Carlo Erba Reagenti, Milan, Italy) as the radical initiator (10% mol/mol). Subsequently, radical polymerization of monomers around the preformed core (PMMA core/monomers weight ratio 1:5) was performed. The reaction was carried out for 3 h at 37 °C, ensuring a constant stirring at 600 rpm. At the end of the polymerization, PMMA core was extracted by three cycles of washing in chloroform (Carlo Erba Reagenti, Milan, Italy) followed by rinsing with water. NCs were freeze-dried overnight and stored at 4 °C until further use. 

### 2.2. Covalent Functionalization

**Figure 1:**
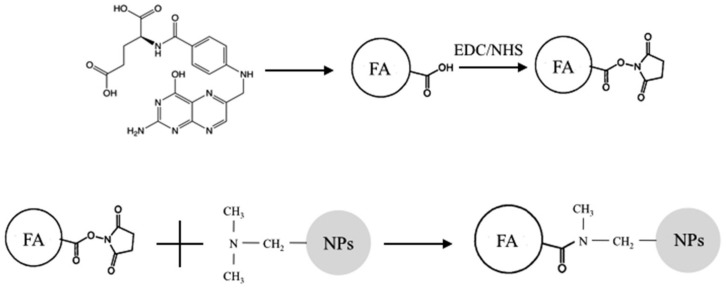
Schematic of the coupling reaction between folic acid (FA) and terpolymer-based nanoparticles (NPs). Activation of carboxylic groups of FA (chemical structure shown at the top right) by EDC/NHS; formation of covalent bonds among activated esters of FA and tertiary amine of DMAEMA monomer units of terpolymer-based nanoparticles.

NCs were covalently functionalized to obtain active targeting towards cancer cells, using folic acid (FA, Mw: 441.4 Da) as the ligand to be bound on the surface of the nanoparticles. First, carboxyl groups of FA were activated to favor the interaction with the amino groups of the material. A 0.045 M FA solution in dimethyl sulfoxide (DMSO) was prepared. Subsequently, 1-Ethyl-3-(3-dimethylaminopropyl)carbodiimide/N-Hydroxysuccinimide (EDC/NHS) were added (FA/EDC molar ratio 1:1, EDC/NHS molar ratio 3:1) and the solution was kept under mild magnetic stirring at room temperature for 18 h to favor the activation of the FA carboxyl groups. At the end of the incubation period, the unreacted EDC/NHS was removed via dialysis against water. Once FA was activated, NCs were dispersed in the solution (NCs/FA weight ratio 1:1) by sonicating them for 1 min at the maximum power using a bath sonicator (Branson 1800) and then kept under magnetic stirring at room temperature for 3 h. Finally, NCs were centrifuged (Mikro 200 Hettich Zentrifugen, Tuttlingen, Germany) at 14,000 rpm for 15 min and washed three times with bidistilled water to remove the excess of FA. A schematic of the reaction is shown in Figure 1. 

Moreover, adsorption of FA was carried out on NCs to have a control for comparison to the covalently functionalized ones. Adsorption was carried out following the same protocol used for the functionalization, using non-activated FA. Subsequently, functionalized NCs and NCs after FA adsorption were dispersed in phosphate buffer solution (PBS) by bath sonication for 1 min at the maximum power, and then kept under vigorous magnetic stirring for 1 h at 70 °C. Samples were centrifuged at 14,000 rpm for 15 min, the supernatant was completely removed and fresh PBS was added. This procedure was repeated three times. The purpose of this treatment was to demonstrate the validity of the process of covalent functionalization. The hypothesis is that after the treatment, FA is no longer present on NCs after adsorption while it continues to be present on covalently functionalized NCs.

The amount of conjugated FA on the NCs (conjugation efficiency) was quantified via absorbance measurements at a wavelength of 280 nm using a spectrophotometer (Shimadzu UV-2100). Specifically, the amount of FA remained in the solution used for the conjugation procedure was analyzed at the end of the incubation period, and the amount of conjugated FA was calculated as the difference between the initial amount of FA in the solution and the amount of FA that remained in the solution at the end of the incubation period. The same method was used to analyze the solutions at the end of the extraction procedure to investigate the presence of FA, if any.

### 2.3. Characterization of the Nanocapsules

#### 2.3.1. Monomer Conversion

To evaluate the final monomer conversion, an aliquot of the polymerization media was collected at the end of the synthesis and analyzed via High Performance Liquid Chromatography (HPLC, Perkin Elmer Series 200, Waltham, MA, USA). The mobile phase consisted of acetonitrile (ACN)/water (80/20 *v/v*) pumped at an isocratic flow rate of 1 mL/min. The analysis was carried out using an Alltima C18 5u (Alltech, 250 mm × 4.6 mm) as a column at a wavelength of 210 nm. The final monomer conversion was evaluated using the following equation:

$$x\left(t\right)=\frac{\left(C_{0}-C_{t}\right)}{C_{0}}$$

where *C_0_* and *C_t_* are the initial and final monomer concentrations in the reactive mass, respectively.

#### 2.3.2. Morphological Analysis

The morphology of the PMMA sacrificial core, as well as the morphology of the NCs before and after the extraction of the core was examined by scanning electron microscopy (SEM JEOL JSM 5600, Jeol, Tokio, Japan). Samples were placed on the SEM sample stage and sputter-coated with gold. Quantitative information regarding the mean size and polydispersity index (PDI) of the NCs was obtained by dynamic light scattering (DLS) using a Zetasizer Malvern nano ZS90 (Malvern, U.K.). The analyses were carried out in triplicate by dispersing the NCs in ethanol, since it represents the most suitable dispersant medium to reduce nanoparticle aggregation, as previously demonstrated by the authors [51].

#### 2.3.3. Physicochemical Analysis 

Fourier-transform infrared spectroscopy (FT-IR) was carried out to obtain information regarding the composition of the NCs using a Spectrum Spotlight Imaging System (Perkin Elmer, Waltham, MA, USA). The same technique was suitable also for the evaluation of the successful removal of the PMMA sacrificial core to form the final hollow nanoparticles, as previously demonstrated by the authors and others [51,52]. Finally, FT-IR analysis was performed to investigate the formation of a stable bond between the polymeric matrix and FA in covalently functionalized NCs.

### 2.4. Cytotoxicity Test

#### 2.4.1. MTT Assay

To evaluate the cytocompatibility of the NCs, the 3-(4,5-dimethylthiazol-2-yl)-2,5-diphenil tetrazolium bromide (MTT) assay was performed using NIH3T3 mouse fibroblast cell line (Cell bank Interlab Cell Line Collection, ST Genova, Italy). NCs were extracted in Dulbecco’s Eagle’s Modified Medium (DMEM) (20 mg/mL) for 24 h at 37 °C. Cells were seeded in 48-well plates at two different densities: 1.2 × 10^4^ cells/cm^2^ and 0.6 × 10^4^ cells/cm^2^ for the 24- and 72-h exposure period, respectively. At 24 h after seeding, culture medium was removed from each well and replaced with 800 μL of fresh culture medium and 200 μL of sterile filtered NC extract to reach the final concentration of 100 μg/mL. No extracts were added to 12 wells used as negative control. Samples were incubated at 37 °C with 5% CO_2_. After 24 and 72 h, 100 μL of MTT solution was added to each well. After an additional 4-h incubation period, medium was replaced with 1 mL DMSO and absorbance was read at 570 nm using an UV spectrophotometer (JASCO; V530, Lecco, Italy).

#### 2.4.2. Propidium Iodide Flow Cytometry Assay

To further evaluate the cytocompatibility of the NCs, Propidium Iodide was used for cell viability assays through FACS analysis. HELA cells and NIH3T3 cells were separately seeded in a 24-well plate. An amount of 5 × 10^4^ and 2.5 × 10^4^ cells per well were seeded for the 24- and 72-h exposure period, respectively. Cells seeded alone were used as negative control. At 24 h after seeding, culture medium was removed from each well and replaced with 800 μL of fresh culture medium and 200 μL of sterile filtered NC extract to reach the final concentration of 100 μg/mL. After 24 and 72 h of culture cells were detached with Trypsin-0.2% EDTA, resuspended in 500 μL of cold 1X PBS (Sigma-Aldrich) and transferred into Falcon tubes for flow cytometry analysis. Then, 10 μL of Propidium Iodide (1:20 in bidistilled water, Sigma-Aldrich) were added and after 5 min cell viability was analyzed through flow cytometry. The experiments were carried out in triplicates for each condition.

### 2.5. Nucleic Acid Adsorption and Release Tests

To verify the possibility to use functionalized NCs for the targeted release of nucleic acids, their ability to load and then release DNA was investigated. Adsorption tests were carried out using a model molecule of DNA (Deoxyribonucleic acid, low molecular weight from salmon sperm). DNA was dissolved in PBS (10 mg/mL), NCs were added to DNA solution (1 mg/mL), vortexed to favor the dispersion, and gently stirred for 2 h. At the end of the incubation period, NCs were collected via centrifugation at 14,000 rpm for 15 min. 

Release tests were performed by adding PBS to the NCs, and vortexing them to favor the dispersion in the release medium. Samples were kept under rotation at 37°C for the entire duration of the release study. Aliquots of the release medium were collected at predetermined time points and fresh PBS was added. Both adsorption and release tests were carried out in triplicate.

The amount of absorbed and released DNA was investigated by High Performance Liquid Chromatography (HPLC, Perkin Elmer Series 200, Waltham, MA, USA). Samples were analyzed using a Synergy 4u Hydro-RP C18 (80 Å, 250 mm × 3.00 mm; Phenomenex, Torrance, CA, USA) as a column and acetonitrile (ACN)/water (7/93 *v/v*), fluxed at 1 mL/min as the internal mobile phase. The injected volume was 50 mL, and the chromatograms acquired at the UV wavelength of 260 nm were used for quantitative analysis. The release kinetics was analyzed using the semi-empirical equation of the power low to describe drug release from polymeric systems:

(1) $$\frac{Mt}{M\infty }=kt^{n}$$

where *Mt* and *M*∞ are the absolute cumulative amounts of drug released at time *t* and infinite time, respectively, *k* is a constant incorporating structural and geometric characteristics of the device, and *n* is the release exponent, indicative of the drug release mechanism [53,54].

### 2.6. Cell Uptake Test

To investigate the ability of functionalized NCs to actively target cancer cells, cell uptake tests were carried out using human cervix epithelioid carcinoma HeLa cells, obtained from the American Type Culture Collection (LGC Promochem, Molsheim, France) having overexpressed FA receptors [55]. Non-functionalized NCs were used as control. NC surface was decorated with a fluorescent dye (Fluorescein, Fluo, molecular weight 332.31 Da) to make samples traceable. Specifically, the carboxyl group of Fluo was activated using EDC/NHS (Fluo/EDC molar ratio 1:1, EDC/NHS molar ratio 3:1) to favor the formation of a stable bond with NPs. Subsequently, NCs were dispersed in Fluo solution (NCs/Fluo molar ratio 1:1) and stirred for four hours. Finally, NCs were centrifuged at 14,000 rpm for 15 min and washed three times with bidistilled water to remove the excess of Fluo.

#### 2.6.1. Flow Cytometric Detection of Functionalized and Non-Functionalized NC Uptake

HeLa cells were seeded at a density of 5 × 10^3^ cells/well in complete DMEM, and plates were incubated at 37 °C with 5% CO_2_ for 24 h to achieve a monolayer. To determine the IRIS Dots uptake and loading contents, cells were incubated with a suspension of NCs (100 μg/mL) in complete DMEM for various incubation times. Cells were then washed twice with phosphate-buffered saline (PBS; Gibco Invitrogen, Milan, Italy) and harvested by trypsinization. Cells were resuspended in PBS and the fluorescence emission of NCs (FL-1) was analyzed on a CyAN ADP flow cytometer using the Summit 4.3 software (Beckman Coulter, Fullerton, CA, USA). About 10,000 cells were measured for each sample. Cell debris signals were removed using morphological criteria.

#### 2.6.2. Confocal Microscopic Detection of Functionalized and Non-Functionalized NC Uptake

To detect the uptake of NCs, HeLa cells were seeded at 10,000 cells cm^−2^ in sterile eight-well μ-slides (Ibidi GmbH, Martinsried, Germany) and allowed to attach for 24 h. Cells were incubated with functionalized and non-functionalized NCs (100 μg/mL) in complete medium for 24 h. Then, the cells were washed three times with PBS and fixed with 4% paraformaldehyde. The cells were mounted with Mowiol (Calbiochem, San Diego, CA, USA). Fluorescence images were obtained with a 510 Carl Zeiss confocal laser microscope using a 63× objective.

### 2.7. Statistics

All the experimental data were expressed as mean ± SD (*n* = 3 unless otherwise specified).

F-test was performed on viability measurements to determine statistically significant differences, if any, in cell viability. Statistically significant differences were designated by a significance criterion (*p* value) below 0.05.

## 3. Results

In this study, previously developed terpolymer-based NCs were functionalized using FA to actively target DNA towards cancer cells.

**Figure 2:**
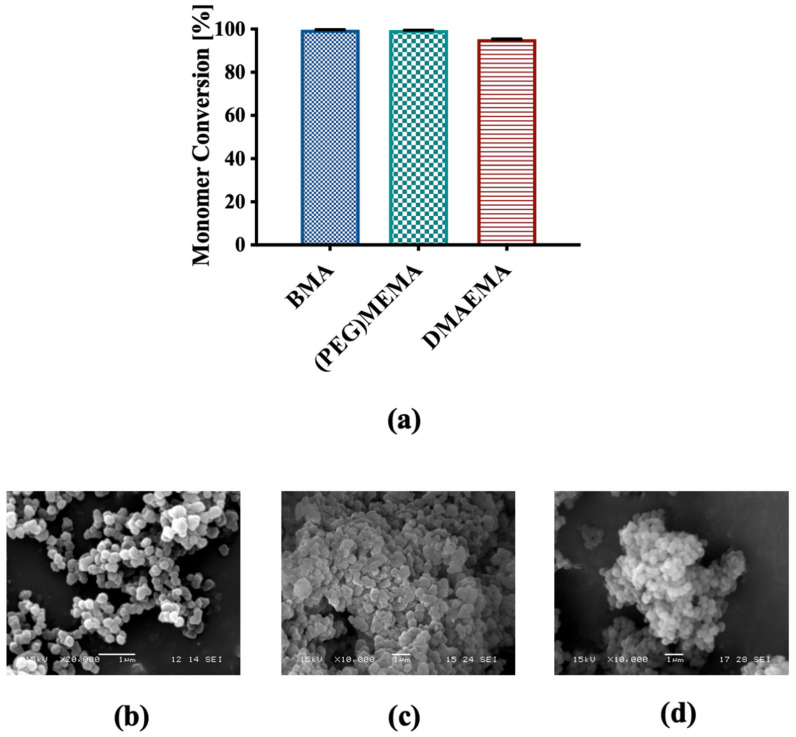
(**a**) Percentage conversion for each monomeric unit; SEM representative micrographs of (**b**) PMMA sacrificial core, (**c**) NCs before and (**d**) after extraction of the core.

Terpolymer-based NCs were characterized for physical and chemical properties before functionalization with FA. The physico-chemical characterization of NCs before functionalization confirmed the results highlighted in a previous published study, supporting the reproducibility of the production process [51]. Briefly, the radical polymerization of the three monomers led to the formation of NCs directly in the reaction phase, with a monomer conversion around 100% for BMA and (PEG)MEMA, and higher than 95% for DMAEMA (Figure 2a). 

SEM analysis highlighted nanometric dimensions and spherical shape of the PMMA core and NCs with a rather homogeneous distribution, and a modest state of aggregation (Figure 2b,c). This was confirmed by DLS measurements that returned an average diameter of the NCs of 224.95 ± 48.43 nm and a PDI of 0.77. Additionally, no significant differences were observed in the morphology of the NCs before and after extraction of the sacrificial core (Figure 2c,d). FT-IR analysis confirmed the previously published results [51], highlighting the presence of the three components in the NCs and confirmed the formation of their hollow structure (data not shown). 

### 3.1. Covalent Functionalization

**Figure 3:**
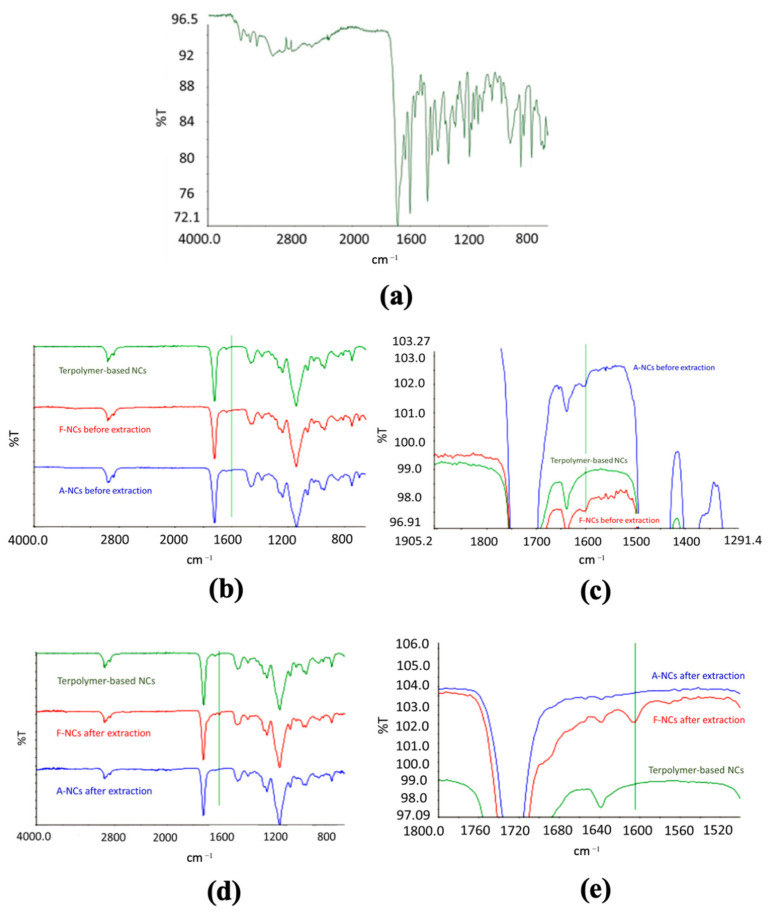
FT-IR analysis. (**a**) Spectrum of folic acid; (**b**) comparison among the spectra of pure terpolymer-based NCs, functionalized NCs, and NCs after adsorption of FA before extraction and (**c**) a magnification; (**d**) comparison between of pure terpolymer-based NCs, functionalized NCs, and NCs after adsorption of FA after removal procedure, and (**e**) a magnification.

NCs were functionalized by covalently binding FA to their surface to obtain active targeting toward cancer cells. The spectra related to pure FA, terpolymer-based NCs, functionalized NCs before and after removal of FA, and NCs after adsorption of FA before and after removal were acquired. FA spectrum showed the diagnostic band around 1600 cm^−1^, which is related to the absorption of the carbonyl group (Figure 3a). 

The same absorption band was evident in functionalized NCs (Figure 3b,c), and it was still present after the removal process (Figure 3d,e), confirming the formation of a stable bond. This result was confirmed by the spectra of NCs after adsorption (Figure 3b,c) and removal (Figure 3d,e) of FA. In fact, the diagnostic band was evident in the spectra of NCs after FA adsorption but it was not present in the spectra of NCs after forced removal of the ligand, confirming the formation of a more stable bond in the case of functionalized NPs. 

The conjugation efficiency was evaluated by analyzing the solutions at the end of the conjugation and at the end of the three washings by absorbance measurements at a wavelength of 280 nm. The analysis returned a conjugation efficiency of 33.6%, corresponding to a mg FA/g NCs of 336, confirming the suitability of the procedure used for a successful formation of a stable bond between the polymer matrix and the activated FA. 

### 3.2. In Vitro Cytotoxicity 

**Figure 4:**
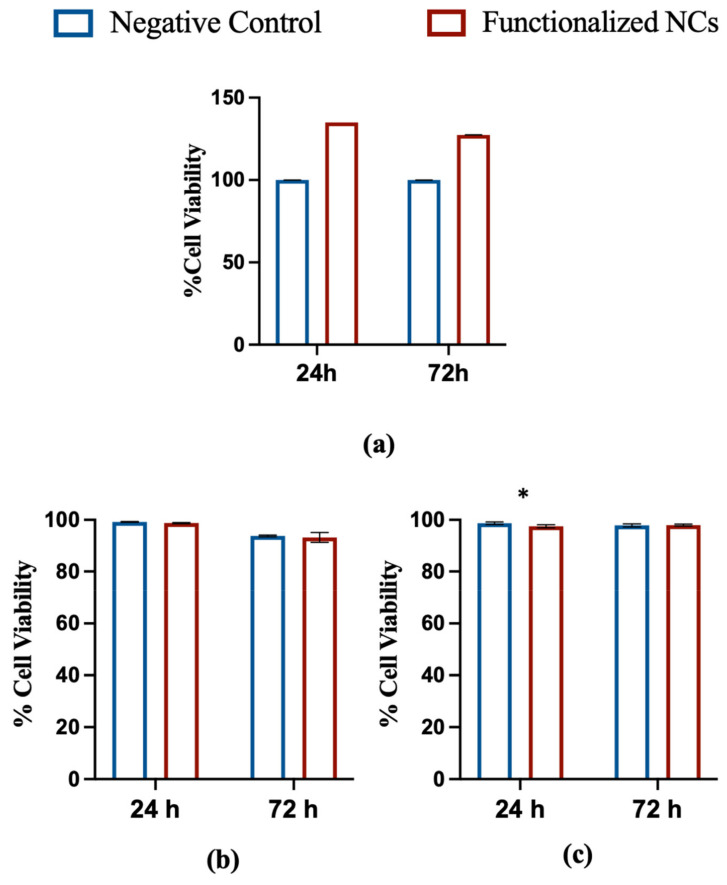
In vitro cytotoxicity tests. (**a**) Cytotoxicity MTT assay of functionalized NCs after 24- and 72-h incubation with NIH3T3 cells. A comparison with negative control is shown; (**b**) Propidium Iodide Flow Cytometry assay of functionalized NCs after 24- and 72-h incubation with HeLa cells. A comparison with negative control is shown; (**c**) Propidium Iodide Flow Cytometry assay of functionalized NCs after 24- and 72-h incubation with NIH3T3 cells. A comparison with negative control is shown (* *p* < 0,05; *t*-test; mean ± SD; *n* = 3).

The cytocompatibility of NCs after covalent functionalization with FA was investigated using both the in vitro MTT assay and the Propidium Iodide Cytometry assay. Specifically, extracts of NCs (corresponding to a concentration of FA of 33.6 μg/mL) were added to NIH3T3 and HeLa cells and the viability after 24 and 72 h of exposure was evaluated. Figure 4 shows that the mitochondrial activity of NIH3T3 cells was not affected by the NC extracts neither after 24- nor 72 h-incubation period. 

The absorbance values acquired by spectrophotometric analysis were subjected to statistical analysis using the F-test, which provided F values smaller than the critical one for all the systems tested, with *p* > 0.05. This result indicates that there are no significant differences between treated cells and negative control. Propidium Iodide Flow Cytometry assay confirmed optimal cell viability at 24 and 72 h for both cell lines. After 24 h, a minimal cytotoxicity was evidenced for NIH3T3 cells only, but no difference was noticed at 72 h versus the negative control, confirming the non-cytotoxicity of the particles after functionalization.

Overall, the obtained results suggest that the modification of the nanoparticles with FA did not affect their cytocompatibility.

### 3.3. Nucleic Acid Adsorption and Release Tests

**Figure 5:**
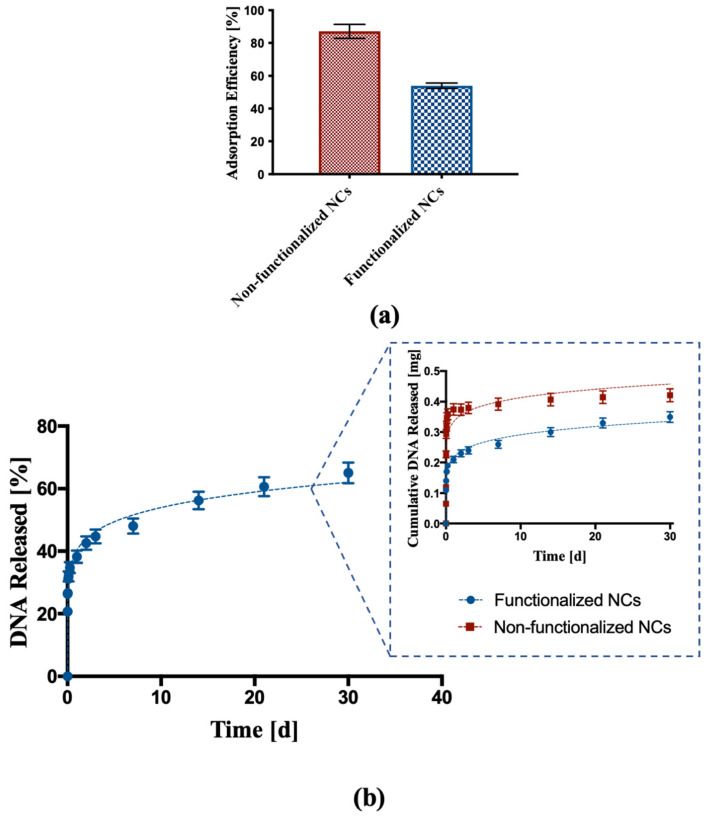
DNA adsorption and release assay. (**a**) DNA adsorption into non-functionalized and functionalized NCs; (**b**) percentage cumulative release of DNA from functionalized NCs. The inset represents a comparison of DNA released from functionalized NCs and non-functionalized NCs expressed as total mass released.

DNA adsorption and release tests were performed on functionalized NCs to determine if the functionalization affected the interactions between polymer and nucleic acid. DNA adsorption test carried out on functionalized NCs showed an adsorption efficiency around 54%, which was lower than the adsorption efficiency of the non-functionalized NCs (87.1%), as shown in Figure 5a.

DNA release from functionalized NCs was also investigated. Figure 5b shows a DNA release profile characterized by an initial burst effect typical of such kind of nanoparticles followed by a constant release with controlled kinetics over time, with almost 60% DNA released over a period of 30 days. The comparison between the DNA released from non-functionalized NCs and functionalized NCs showed for the latter a reduction in the initial burst effect and a more linear release profile over time.

### 3.4. Cell Uptake

**Figure 6:**
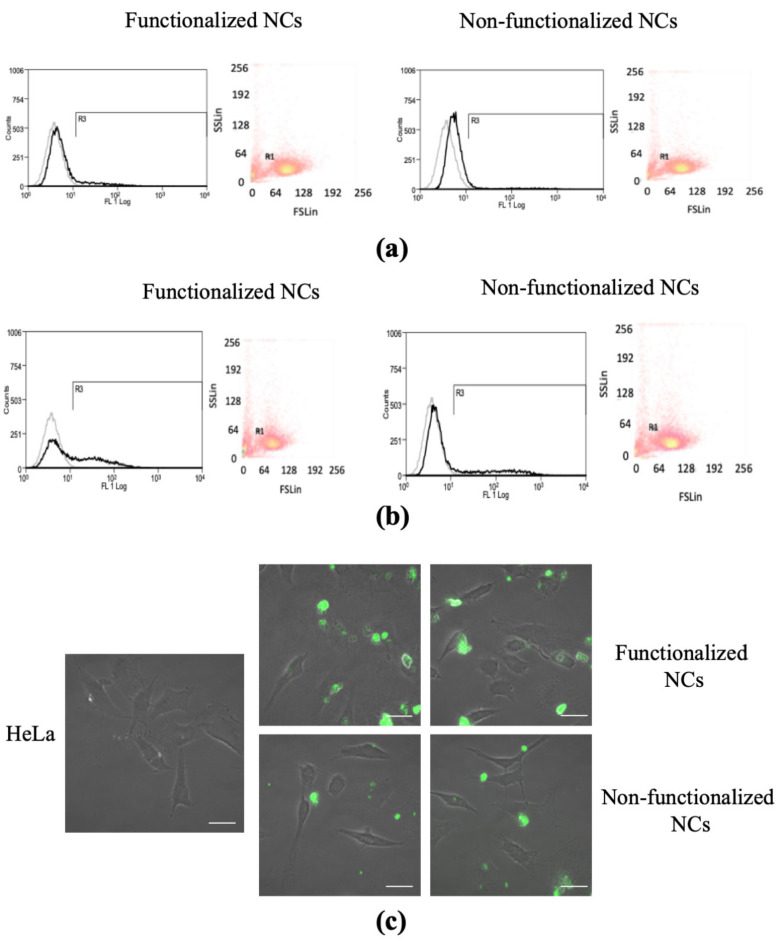
Cell uptake test. Fluorescence-activate cell sorting (FACS) analysis representing the dot plots of physical cell parameters and histogram plots of fluorescence emitting HeLa cells after (**a**) 6-h and (**b**) 24-h incubation with functionalized and non-functionalized NCs. Black histograms represent cells stained with 100 μg/mL^−1^ NCs. Gray histograms represent unstained control cells; (**c**) confocal microscopy images of HeLa cells after 24 h of incubation with 100 μg mL^−1^ functionalized and non-functionalized NCs. Scale bar 20 μm.

Cell uptake tests were carried out on covalently functionalized, fluorescinated NCs using HeLa cells to investigate their ability to actively target cancer cells overexpressing FA receptors. Non-functionalized, fluorescinated NCs were used as control. In a first set of cellular uptake experiments, HeLa cells were incubated with 100 μg/mL of either functionalized or non-functionalized NCs in complete medium for various incubation times (6 and 24 h) at 37 °C. After the indicated time, the cells were trypsinized and cellular uptake of the two types of NCs was determined by flow cytometry. Uptake of NCs began as early as 6 h after incubation and increased at 24 h, thus revealing that NCs uptake was time-dependent (Figure 6a,b). 

Interestingly, this analysis highlighted a higher percentage of emitted fluorescence in the case of covalently functionalized NCs than control NCs, thus suggesting that FA-functionalization was effective in augmenting internalization rate (Figure 6 and Table 2).

In a further set of experiments, HeLa cells were plated in sterile eight-well μ-slides and incubated for 24 h with NCs, then the cells were washed twice to remove the excess NCs passively adsorbed on the cell surface and imaged by confocal microscopy. Both functionalized and non-functionalized NCs entered the cells and in 24 h they could be localized either near the cell membrane or distributed inside the cell cytoplasm (Figure 6c). In agreement with the quantitative FACS analysis of cellular uptake (Figure 6a,b), increased internalization was observed in the case of functionalized NCs compared to the non-functionalized ones, confirming a higher ability of the functionalized NCs to be uptaken by cells overexpressing FA receptors (Figure 6c).

## 4. Discussion

The radical polymerization of BMA, DMAEMA, and PEG was carried out to synthesize innovative terpolymer-based NPs with properties suitable for the encapsulation of nucleic acids. The development of a three component-based copolymer was triggered by the need to develop a drug delivery system capable of enhancing the loading and the release of DNA, thanks to the combination of the properties of each element. Specifically, the acrylic polymer PBMA is well known for its biocompatibility, and it has been used to fabricate platforms for gene delivery [56,57,58]., Given its chemical properties and low toxicity, pDMAEMA has been largely utilized as non-viral gene delivery system [59,60]. Finally, PEG chains were introduced to make the surface of the NPs more hydrophilic and minimize the protein absorption, thus reducing the reticuloendothelial clearance [61,62].

The template polymerization of the three distinct monomers around a preformed polymeric core led to the formation of hollow NCs with a spherical shape and nanometric dimensions. FT-IR analysis highlighted the success of the copolymerization, confirming the HPLC results that showed a monomer conversion greater than 95% for all the monomers. Furthermore, the analysis pointed out the removal of the PMMA core that led to the formation of the hollow structure of the NCs. 

To obtain active targeting towards cancer cells, NCs were covalently functionalized with FA, and FT-IR analysis was performed to verify the formation of a stable bond. Indeed, FA is an essential molecule in DNA synthesis pathway which is highly needed for cancer cell duplication. As a consequence, many cancer cells overexpress folate receptors higher than normal cells, and this fact is the basis of the folate targeting strategy [47]. The comparison between the spectra acquired on functionalized NCs and control NCs after adsorption of FA showed the presence of the diagnostic band around 1600 cm^−1^ due to the absorption of the carbonyl group of the FA in both samples before the removal of the ligand under drastic conditions. The FA diagnostic band was still evident in the spectra of functionalized NCs after the removal procedure, while it was not present anymore in the spectra of NCs after FA adsorption and subjected to the same FA removal procedure, suggesting that the covalent functionalization was successful. This might be due to the formation of a stable bond between the activated carbonyl group of FA and the amino group of the pDMAEMA, while the interactions occurring between FA and NCs during the adsorption procedure were not stable enough, thus leading to the removal of the ligand after repeated washings. 

In view of a possible use of these nano-systems for the delivery of a nucleic acid to treat pathologies such as cancer, DNA with a low molecular weight was selected as the model molecule to be encapsulated into NPs. NCs showed a good adsorption efficiency, mainly due to the formation of hydrogen bonds between NCs and DNA, in addition to the presence of pDMAEMA, which provides amino groups to the terpolymer that favor the molecular interaction [63,64,65]. Interestingly, the adsorption efficiency of the functionalized NCs was lower than the adsorption efficiency of the non-functionalized ones. This might be due to part of the functional groups of the pDMAEMA interacting with the carboxyl group of the FA, leading to less amino groups available for the interaction with the DNA. Despite this, the capability of the NCs to release DNA with a controlled trend was not affected by the lower adsorption efficiency. In fact, the release assay carried on NCs after covalent functionalization highlighted a more controlled kinetics if compared to the one of non-functionalized NCs, with a reduction of the initial amount of DNA released and a more constant release over time. Analyzing the release profile more in depth, the initial burst effect was probably due to the DNA weakly bound to the surface of the NCs and/or to the polymeric matrix immediately below the surface. In the subsequent phase, the DNA release slowed down and it was prolonged over time. The more controlled release kinetics might be due to the presence of FA on the surface of the NPs, forming an additional layer through which DNA has to diffuse before being released. In addition, after 30 days, only 60% of the absorbed DNA was released, suggesting that the nucleic acid in the inner part of the cavity needs more time to diffuse through the polymer and be delivered. Overall, terpolymer-based NCs, thanks to their composition and hollow structure, represent a delivery system capable of enhancing the amount of encapsulated DNA, as well as leading to a more prolonged release over time. Indeed, many polymeric drug delivery systems have been investigated for the delivery of nucleic acid; however, the loading of DNA is often lower than 50% with a fast release in the order of hours [66,67,68,69]. 

In addition, release kinetics was analyzed using the semi-empirical equation of the power law to describe drug release from polymeric nanoparticles. The fitting accuracy R^2^ and the n, k values are shown in Table 3. 

Specifically, a high degree of correlation was obtained for both functionalized and non-functionalized NCs, with a regression coefficient R^2^ = 0.989 for the functionalized NCs. The R^2^ was lower for the non-functionalized NCs (R^2^ = 0.818), and this can be attributed to the faster release of DNA in the very first hours. Moreover, the release order value *n* < 0.4 suggested a release mechanism governed by Fickian diffusion, which is typical of spherical nanoparticles [54]. This suggests that there is no influence of macromolecular relaxation phenomena as expected for cross-linked nanoparticles. 

A wide selection of constructs which target FA receptors on cancer cells have been developed. Although NP-based carriers allowed higher loading capacity, cytotoxicity is a frequent side effect [70].

The cytotoxicity of NCs after functionalization was evaluated via MTT assay. The test did not show any toxic effect on NIH3T3 cells, confirming that the presence of FA did not affect the cytocompatibility of the NPs, thus supporting the potential use of the terpolymer-based nanocarriers for drug delivery applications. 

Finally, preliminary cell uptake tests carried out on cells over-expressing FA receptors highlighted an increased ability of the functionalized NCs to actively target the cells than the non-functionalized ones. HeLa cells are well known to have folic acid receptors on their surface, making them suitable cells to test uptake of FA-functionalized nanoparticles [55]. Our preliminary test on this cell line supports the idea that presence of targeting moieties on the surface of the nanoparticles favors cell uptake due to a ligand-receptor-based mechanism, thus opening up the possibility to more effective therapies. In addition to this, direct molecular interaction of FA with cellular membranes of cancer cells has been previously demonstrated not to be limited to this specific receptor-mediated contact, highlighting the advantages of rational design of nanosystems as well as the possible involvement of direct molecular interactions of FA with cellular membranes, not limited to specific receptor recognition, in the mechanisms of their endocytosis [71]. 

Overall, these results are very promising and suggest that terpolymer-based NCs could be a potential innovative system for the targeted delivery of nucleic acids to cancer cells. Thanks to their cavity, NCs allows for a modulation of the release over time, and the modification of their surface helps targeting the cells of interest. Future studies will be focused on further investigation of the NC ability to target specific cancer cells (such as ovarian cancer cells). Moreover, functionalized NCs will be tested in vivo on a murine model of ovarian cancer, and their ability to accumulate in the diseased region upon systemic injection will be investigated using imaging techniques. 

## 5. Conclusions

In this study, terpolymer-based nanocapsules were developed via free radical polymerization of three distinct monomers around a preformed sacrificial core. The NCs were loaded with DNA and functionalized with FA to obtain active targeting towards cancer cells via a ligand-receptor-based mechanism. The NCs were able to encapsulate the DNA and then release it over time in a controlled way, thanks to the hollow structure of the particles that favored a modulation of the release. The modification of the NC surface led to a carrier able to target HeLa cells overexpressing folate receptors, favoring cell uptake via a ligand-receptor-based mechanism. Overall, these results are very promising and suggest that terpolymer-based NCs could be a potential innovative system for the targeted delivery of nucleic acids to cancer cells, opening up the possibility of more effective therapies.

## Figures and Tables

**Table 1:** Chemical structures of the monomers used for the synthesis of the sacrificial core (MMA), nanocapsules (BMA, DMAEMA, (PEG)MEMA), and cross-linker (TRIM).

Monomer	Chemical Structure
Methyl methacrylate (MMA)	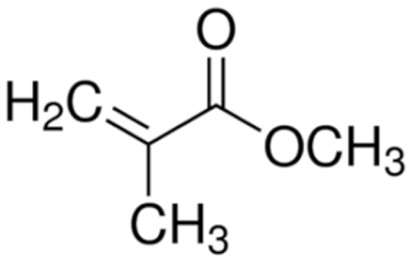
n-Butyl methacrylate (BMA)	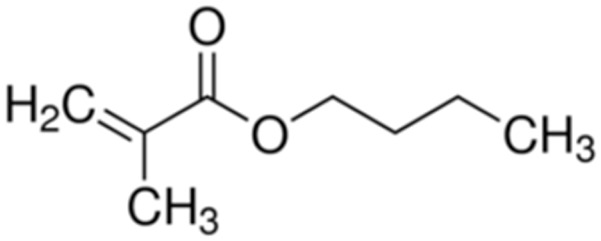
2-(dimethylamino)ethyl methacrylate (DMAEMA)	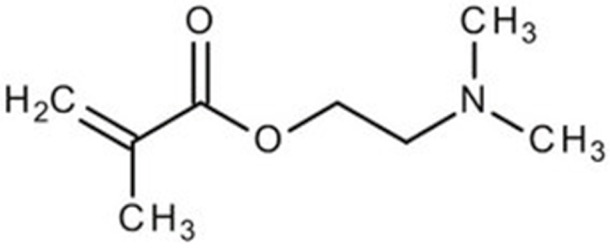
Poly(ethylene glycol) methyl ether methacrylate (PEG)MEMA	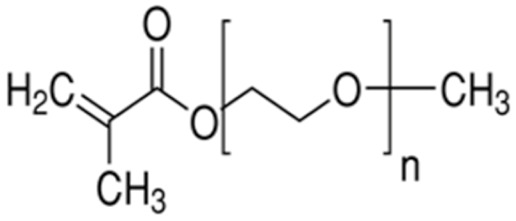
Trimethylpropane trimethacrylate (TRIM)	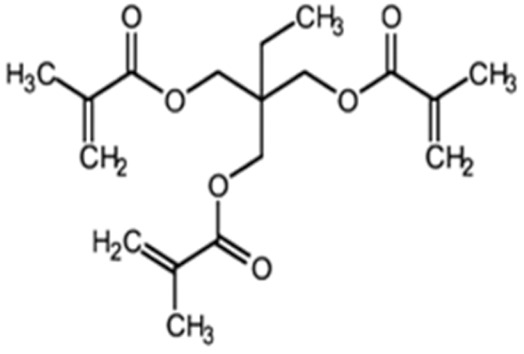

**Table 2:** FACS analysis. Percentage of HeLa cells emitting fluorescence at the different time points.

	Functionalized NCs	Non-Functionalized NCs
	% Count	% Count
6 h	12.45	6.69
24 h	40.23	21.77

**Table 3:** Power law parameters (n, K) and fitting accuracy R^2^ for DNA release from functionalized and non-functionalized NCs.

	R^2^	n	K
Functionalized NCs	0.989	0.117 to 0.139	0.210 to 0.223
Non-functionalized NCs	0.818	0.052 to 0.133	0.298 to 0.369

## Data Availability

The raw data required to reproduce these results are available from the corresponding author upon reasonable request.
